# Effect of blood lipids and lipid-lowering therapies on osteoarthritis risk: A Mendelian randomization study

**DOI:** 10.3389/fmed.2022.990569

**Published:** 2022-11-11

**Authors:** Zhaolun Wang, Mengyuan Liu, Yixin Zhou, Hongyi Shao, Dejin Yang, Yong Huang, Wang Deng

**Affiliations:** ^1^Department of Orthopedic Surgery, Beijing Jishuitan Hospital, Fourth Clinical College of Peking University, Beijing, China; ^2^State Key Laboratory of Cardiovascular Disease, Department of Cardiology, Fuwai Hospital, National Center for Cardiovascular Diseases, Chinese Academy of Medical Sciences and Peking Union Medical College, Beijing, China

**Keywords:** osteoarthritis, statins, Mendelian randomization, ezetimibe, blood lipid

## Abstract

**Background:**

We aimed to investigate the effects of blood lipids and lipid-lowering agents on osteoarthritis (OA) risk.

**Materials and methods:**

We performed Mendelian randomization (MR) analyses to estimate the causal effect of blood low-density lipoprotein cholesterol (LDL-C), high-density lipoprotein cholesterol (HDL-C), and triglyceride (TG) levels on knee and hip OA. Single nucleotide polymorphisms (SNPs) were selected from large genome-wide association studies (GWASs) of individuals of European ancestry as genetic instruments for blood lipid levels. The associations of selected genetic instruments with knee and hip OA were estimated in a recent GWAS of the UK Biobank and arcOGEN datasets. Univariate and multivariate MR analyses were performed to detect and adjust for potential pleiotropy. Furthermore, genetic instruments in *HMGCR, NPC1L1*, and *PCSK9* regions were used to mimic LDL-C-lowering effects of statin, ezetimibe, and evolocumab, respectively.

**Results:**

Genetically determined LDL-C increments led to reduced risks of both knee OA (OR = 0.91 per 1-SD increment, 95% CI: 0.86–0.95, *P* = 6.3 × 10^−5^) and hip OA (OR = 0.92, 95% CI: 0.85–0.99, *P* = 0.027). Multivariate MR analysis proved that the effect was independent of HDL-C, TG, and body mass index. TG increment was associated with reduced risks of hip OA in the univariate MR analysis; however, this was not supported by the multivariate MR analysis. Genetically proxied LDL-C-lowering effects of statins are related to increased risks of knee OA but not hip OA.

**Conclusions:**

The findings suggested that LDL-C increments have independent protective effects on both knee and hip OA. LDL-C-lowering effects of statins may increase the risk of knee OA.

## Introduction

Osteoarthritis (OA) is the most common form of arthritis; it affects more than 5% of people worldwide, and its prevalence is growing (1). OA is characterized by articular cartilage degeneration, chronic pain, joint deformities, and eventual disability (2). Although the etiology of OA is not well-understood, it is considered a metabolic syndrome-associated disease rather than a purely age- or weight-related disease (3). Experimental studies and a recent meta-analysis of observational studies showed that dyslipidemia is involved in OA pathophysiology (4, 5). However, the causal effect of blood lipid profile, particularly low-density lipoprotein cholesterol (LDL-C), on the risk of developing OA remains unclear.

Statins, inhibitors of 3-hydroxy-3-methylglutaryl-coenzyme A reductase (HMGCR), are the most frequently prescribed cholesterol-lowering drugs. These drugs are recommended as first-line therapy to reduce the risk of atherosclerotic cardiovascular disease (ASCVD) (6). To date, the effects of statins on OA have aroused great interest from researchers; however, results are conflicting and vary from reduced risk to no effect or even an increased risk of OA (3, 7). Nevertheless, the current evidence is limited to observational studies and is inevitably affected by confounding factors, making it difficult to clarify the causal relationship. In recent years, in addition to statins, newer lipid-lowering agents acting on different mechanisms, including ezetimibe or anti-proprotein convertase subtilisin/kexin type 9 (PCSK9) monoclonal antibodies (evolocumab), have been widely used to achieve LDL-C targets in the secondary prevention of ASCVD (8). However, the effects of these drugs on OA have not been reported.

Therefore, we aimed to use a Mendelian randomization (MR) approach to investigate the effect of blood lipid profiles and lipid-lowering agents on OA risk. Because MR employs genetic variants associated with the target of cholesterol-lowering agents, which are random with respect to potential confounding factors, our study will help clarify the causal relationship among blood lipids, lipid-lowering agents, and OA.

## Materials and methods

This study was performed according to the guidelines of the Strengthening the Reporting of Observational Studies in Epidemiology using Mendelian Randomization (STROBE-MR) (9).

### Genetic instruments for blood lipids

Single nucleotide polymorphisms (SNPs) for plasma LDL-C, high-density lipoprotein cholesterol (HDL-C), and triglyceride (TG) were selected as instrumental variables. Data on these variables were obtained from the genome-wide association studies (GWASs) of the Global Lipids Genetics Consortium (10), which included 188,578 individuals with European ancestry and excluded those receiving lipid-lowering treatment. The included SNPs need to be significantly associated with the trait at the genome-wide level (*P* < 5 × 10^−8^) and independent of each other (*r*^2^ < 0.001). Among the included SNPs, 81, 89, and 55 were associated with LDL-C, HDL-C, and TG, respectively (Supplementary Tables S1–S3).

### Assessment of knee and hip OA

The associations between the selected genetic instruments and knee and hip OA were estimated in a recent GWAS meta-analysis of the UK Biobank and Arthritis Research UK Osteoarthritis Genetics (arcOGEN) datasets (77,052 cases and 378,169 controls) (11), which also included individuals with European ancestry. The UK Biobank is a cohort based on 22 assessment centers in the UK that includes 500,000 participants aged 40–69 years and recruited from 2006 to 2010 (12). The diagnosis of hip and knee OA was based on self-reports and hospital records in the UK Biobank. The arcOGEN dataset includes unrelated UK-based knee and hip OA cases from the ArcOGEN Consortium (13). Knee and hip OA were diagnosed if the individual underwent total joint replacement or had radiographic evidence of OA (Kellgren–Lawrence grade ≥2).

### Two-sample MR

We conducted two-sample MR analysis using the “TwoSampleMR” package in the R software (version 4.1.2). First, we performed a harmonization process to ensure that the effect alleles of SNPs were the same for exposure and outcome. Palindromic SNPs were aligned if the minor allele frequency was <0.3. As a result, two LDL-C SNPs and three HDL-C SNPs were excluded because they were palindromic with intermediate allele frequencies. To estimate the individual effect of each SNP, the Wald ratio was calculated by dividing the SNP-outcome association by the SNP-exposure association. We primarily estimated the causal effect of blood lipids on OA using the random-effect inverse variance-weighted (IVW) method. Estimates of causal effects were reported as odds ratios (ORs) per one standard deviation (SD) increase in LDL-C, HDL-C, and TG. The weighted median, MR-Egger regression, weighted mode, and Mendelian Randomization Pleiotropy RESidual Sum and Outlier (MR-PRESSO) outlier-corrected methods were used for additional sensitivity analyses. The weighted median method can provide an unbiased estimate of the causal effect even if half of the SNPs exhibit pleiotropy (14). The MR-Egger method adds a non-zero intercept to allow directional horizontal pleiotropy (15). It makes the assumption that horizontal pleiotropic effects are independent of SNP-exposure effects, which is also known as the InSIDE assumption. In addition, the MR-Egger regression intercept value was used to estimate the degree of horizontal pleiotropic effects. The weighted mode method groups SNPs according to the similarity of their effects and estimates the causal effect based on the largest cluster of SNPs. Therefore, it can provide an unbiased causal effect estimate as long as the largest cluster of SNPs is valid (16). The MR-PRESSO method can reduce the heterogeneity of the estimate by correcting the effects of outlier SNPs (17). We conducted MR-PRESSO analysis using the MR-PRESSO R package and set the number of distributions to 10,000 and the significance threshold to 0.05. For additional sensitivity analyses, we used forest plots for visual inspection of potential pleiotropy. Cochran's Q statistics were calculated to assess the extent of heterogeneity, with *P*-values < 0.05 indicating significant heterogeneity. The causal direction between exposure and outcome was determined using the Steiger test, which compares the extent of outcome variance and exposure variance explained by instrumental variables (16).

### Multivariable MR

As the included instrument SNPs may be associated with multiple lipid fractions and body mass index (BMI), we performed multivariable MR to estimate the independent effect of each lipid. Multivariable MR analysis was conducted using the IVW method. Instrumental SNPs for BMI were selected from a GWAS meta-analysis of European ancestry conducted by the Genetic Investigation of ANthropometric Traits (GIANT) Consortium (18) (Supplementary Table S4).

### Estimating the effect of lipid-lowering therapy on OA risk

Three sets of SNPs within the *HMGCR*, Niemann-Pick C1-Like 1 (*NPC1L1*), and *PCSK9* genes were used to mimic the LDL-C-lowering effect of statins, ezetimibe, and evolocumab, respectively, as used in previous studies (10, 19–23) (Supplementary Table S5). Because some SNPs were not completely independent (*r*^2^ value for linkage disequilibrium < 0.3), we estimated the causal effect of lipid-lowering therapy on OA using the random-effect IVW method that accounted for the correlation among variants, provided by the Mendelian Randomization R package (24). The linkage disequilibrium matrix for SNPs was extracted from the European 1,000 genome data (25).

## Results

### Causal effects of blood LDL-C on OA risk

**Figure 1:**
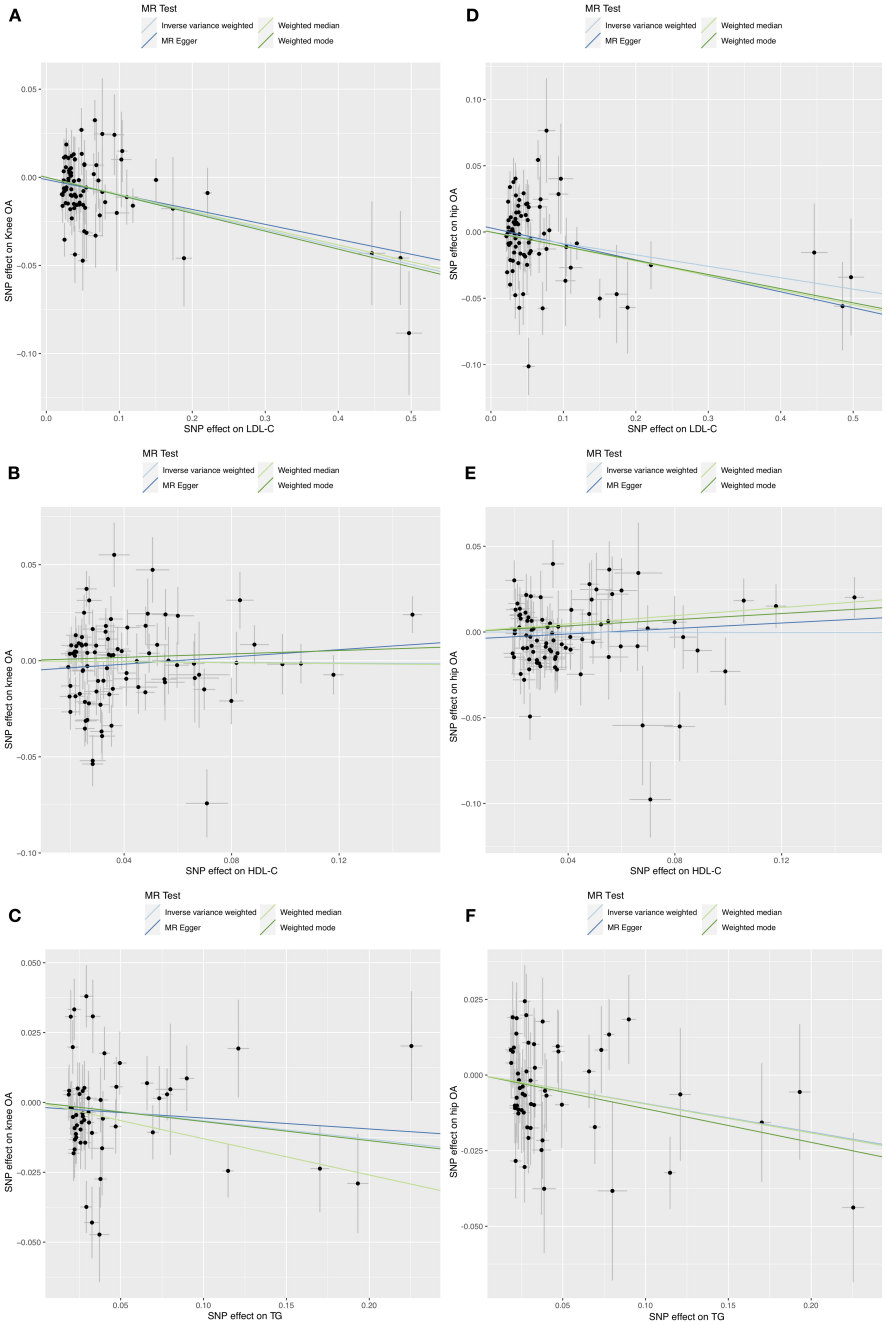
Scatter plots of MR analyses for the causal effect of LDL-C, HDL-C, and TG on knee **(A–C)** and hip **(D–F)** OA risk.

IVW MR suggested that LDL-C increment was associated with reduced risks of knee OA (OR = 0.91, 95% CI: 0.86–0.95, *P* = 6.3 × 10^−5^) and hip OA (OR = 0.92, 95% CI: 0.85–0.99, *P* = 0.027). The MR-Egger, weighted median, weighted mode, and MR-PRESSO outlier-corrected methods yielded similar results (Table 1). The Cochrane Q statistic suggested significant heterogeneity (knee OA, Q = 113.77, *P* = 0.005; hip OA, Q = 180.24, *P* < 0.001). The MR-Egger regression showed no evidence of horizontal pleiotropy for knee OA (Egger intercept = −0.001, *P* = 0.589) or hip OA (Egger intercept = 0.003, *P* = 0.410) (Figures 1A,D). Visual inspection of the funnel plots revealed no signs of horizontal pleiotropy (Supplementary Figures S1, S2). The MR-PRESSO analysis identified one outlier and three outliers for knee and hip OA, respectively. However, these outliers did not influence the effect estimates for knee OA (MR-PRESSO distortion test *P*-value = 0.846) or hip OA (*P* = 0.708). In addition, the Steiger test demonstrated a causal relationship between exposure and outcome (*P* < 0.001).

**Table 1:** Univariate Mendelian randomization analysis results.

**Exposure**	**Outcome**	**Methods**	**Number of SNPs**	**OR (95% CI)**	***P*-value**
LDL-C	Knee OA	Inverse variance weighted	79	0.91 (0.86–0.95)	6.3 × 10^−5^
		MR Egger	79	0.92 (0.86–0.99)	0.022
		Weighted median	79	0.91 (0.85–0.97)	0.003
		Weighted mode	79	0.90 (0.85–0.96)	9.4 × 10^−4^
		MR-PRESSO outlier-corrected	79 (1 outlier SNP)	0.91 (0.87–0.95)	1.3 × 10^−4^
LDL-C	Hip OA	Inverse variance weighted	79	0.92 (0.85–0.99)	0.027
		MR Egger	79	0.89 (0.79–0.99)	0.038
		Weighted median	79	0.90 (0.83–0.97)	0.006
		Weighted mode	79	0.90 (0.84–0.96)	0.003
		MR-PRESSO outlier-corrected	79 (3 outlier SNPs)	0.91 (0.85–0.97)	0.006
HDL-C	Knee OA	Inverse variance weighted	86	0.99 (0.90–1.09)	0.835
		MR Egger	86	1.10 (0.93–1.30)	0.277
		Weighted median	86	0.99 (0.90–1.08)	0.793
		Weighted mode	86	1.05 (0.96–1.14)	0.309
		MR-PRESSO outlier-corrected	86 (6 outlier SNPs)	1.01 (0.94–1.09)	0.771
HDL-C	Hip OA	Inverse variance weighted	86	1.00 (0.92–1.08)	0.968
		MR Egger	86	1.08 (0.93–1.26)	0.313
		Weighted median	86	1.13 (1.02–1.25)	0.025
		Weighted mode	86	1.09 (0.98–1.22)	0.112
		MR-PRESSO outlier-corrected	86 (2 outlier SNPs)	1.02 (0.95–1.10)	0.585
TG	Knee OA	Inverse variance weighted	55	0.94 (0.86–1.02)	0.135
		MR Egger	55	0.96 (0.84–1.11)	0.584
		Weighted median	55	0.88 (0.80–0.96)	0.005
		Weighted mode	55	0.93 (0.86–1.02)	0.136
		MR-PRESSO outlier-corrected	55 (3 outlier SNPs)	0.93 (0.87–1.00)	0.072
TG	Hip OA	Inverse variance weighted	55	0.91 (0.84–0.98)	0.017
		MR Egger	55	0.91 (0.80–1.03)	0.149
		Weighted median	55	0.91 (0.81–1.02)	0.106
		Weighted mode	55	0.89 (0.80–1.00)	0.053
		MR-PRESSO outlier-corrected	55 (0 outlier SNPs)	0.91 (0.84–0.98)	0.021

OA, Osteoarthritis; SNP, single nucleotide polymorphisms; LDL-C, low-density lipoprotein cholesterol; HDL-C, high-density lipoprotein cholesterol; TG, triglyceride; MR-PRESSO, Mendelian Randomization Pleiotropy RESidual Sum and Outlier.

### Causal effects of blood HDL-C on OA risk

IVW MR revealed no evidence of the association between HDL-C levels and OA risk (Table 1). The Cochrane Q statistic suggested significant heterogeneity (knee OA: Q = 273.34, P < 0.001; hip OA: Q = 142.05, P < 0.001). The MR-Egger regression showed no evidence of horizontal pleiotropy for knee OA (Egger intercept = −0.006, *P* = 0.157) or hip OA (Egger intercept = −0.004, *P* = 0.223) (Figures 1B,E). Visual inspection of funnel plots revealed no horizontal pleiotropy (Supplementary Figures S3, S4). The MR-PRESSO analysis revealed six outliers and two outliers for knee and hip OA, respectively. However, these outliers did not influence the effect estimates for knee OA (*P* = 0.132) and hip OA (*P* = 0.201).

### Causal effects of blood TG on OA risk

IVW MR revealed that TG increment was associated with reduced risks of hip OA (OR = 0.91, 95% CI: 0.84–0.98, *P* = 0.017) but not knee OA (OR = 0.94, 95% CI: 0.86–1.02, *P* = 0.135) (Table 1). The effect of TG on hip OA was reproduced using the MR-PRESSO method (OR = 0.91, 95% CI: 0.84–0.98, *P* = 0.021). Weighted mode MR also showed a trend toward reduced risks of hip OA (OR = 0.89, 95% CI: 0.80–1.00, *P* = 0.053). However, the MR-Egger regression and weighted median MR did not show any association. The Cochrane Q statistic suggested significant heterogeneity in the association between TG and knee OA (Q = 125.11, *P* < 0.001) but not between TG and hip OA (Q = 64.85, *P* = 0.148). The MR-Egger regression showed no evidence of horizontal pleiotropy for knee OA (Egger intercept = −0.002, *P* = 0.635) and hip OA (Egger intercept=0, *P* = 0.994) (Figures 1C,F). Visual inspection of funnel plots revealed no horizontal pleiotropy (Supplementary Figures S5, S6). The MR-PRESSO analysis identified three outliers for knee OA. However, these outliers did not influence the effect estimates (*P* = 0.930). In addition, the Steiger test demonstrated a causal relationship between exposure and outcome (*P* < 0.001).

### Multivariable MR

**Figure 2:**
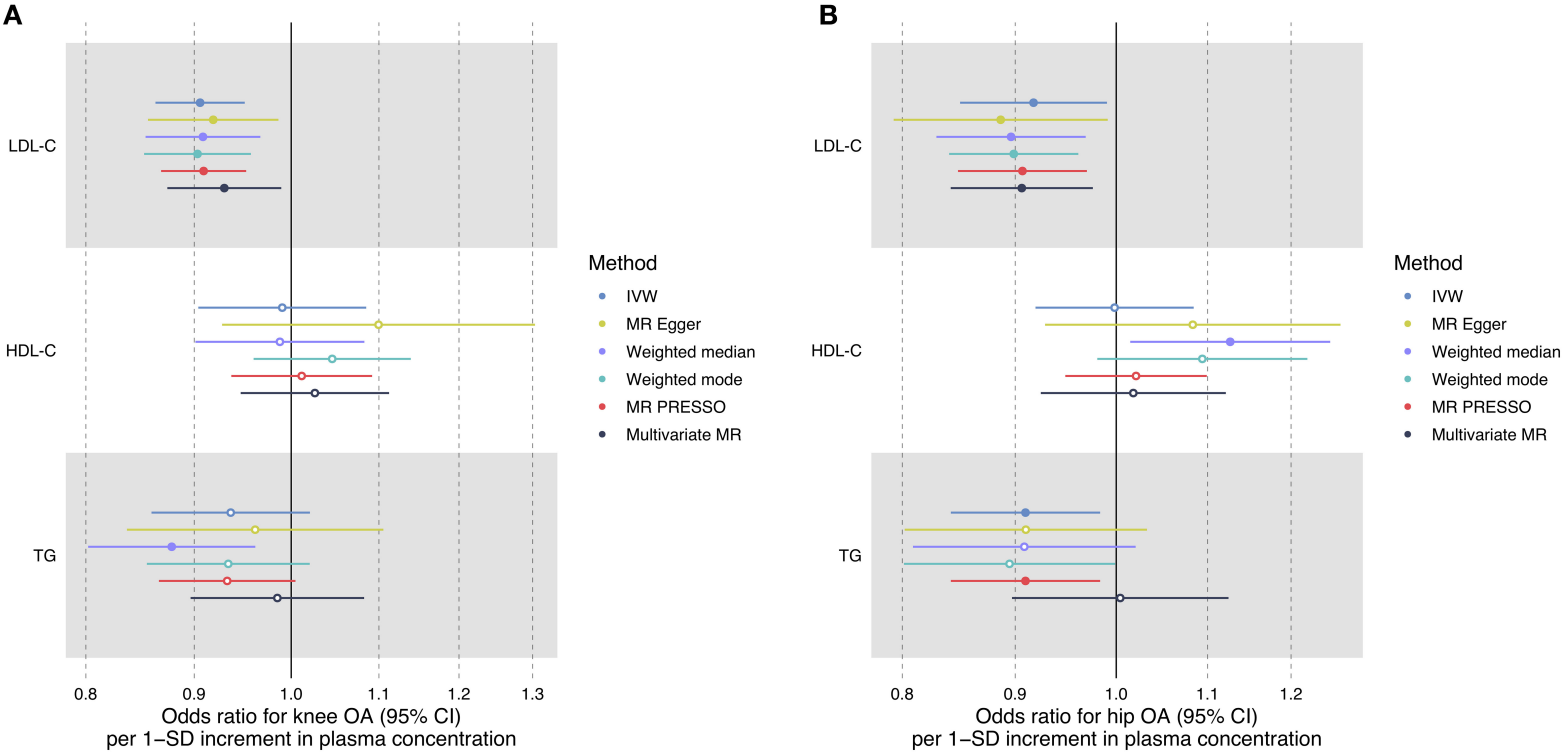
Forest plot comparing causal effect estimates of serum lipid levels on knee **(A)** and hip **(B)** OA risk using univariate and multivariable MR analyses. Odds ratios (ORs) with 95% confidence intervals (CIs) are scaled to 1-SD increment in blood lipid level.

Multivariable MR revealed a protective effect of LDL-C increment on the risk of knee OA (OR = 0.93, 95% CI: 0.87–0.99, *P* = 0.021) and hip OA (OR = 0.91, 95% CI: 0.84–0.98, *P* = 0.009) independent of HDL-C, TG, and BMI (Table 2). The estimated OR was comparable to that obtained using univariate MR analyses (Figure 2). Multivariable MR analysis revealed that neither HDL-C nor TG level was associated with OA risk.

**Table 2:** Multivariate Mendelian randomization analysis results.

**Exposure**	**Outcome**	**Number of SNPs**	**OR (95% CI)**	***P*-value**
LDL-C	Knee OA	38	0.93 (0.87–0.99)	0.021
HDL-C	Knee OA	50	1.03 (0.95–1.11)	0.532
TG	Knee OA	27	0.99 (0.90–1.08)	0.755
BMI	Knee OA	360	2.14 (1.95–2.34)	6.4 × 10^−61^
LDL-C	Hip OA	38	0.91 (0.84–0.98)	0.009
HDL-C	Hip OA	50	1.02 (0.92–1.12)	0.717
TG	Hip OA	27	1.00 (0.90–1.12)	0.942
BMI	Hip OA	360	1.54 (1.38–1.72)	5.3 × 10^−15^

OA, Osteoarthritis; SNP, single nucleotide polymorphisms; LDL-C, low-density lipoprotein cholesterol; HDL-C, high-density lipoprotein cholesterol; TG, triglyceride; BMI, body mass index.

### Causal effects of lipid-lowering therapy on OA risk

**Figure 3:**
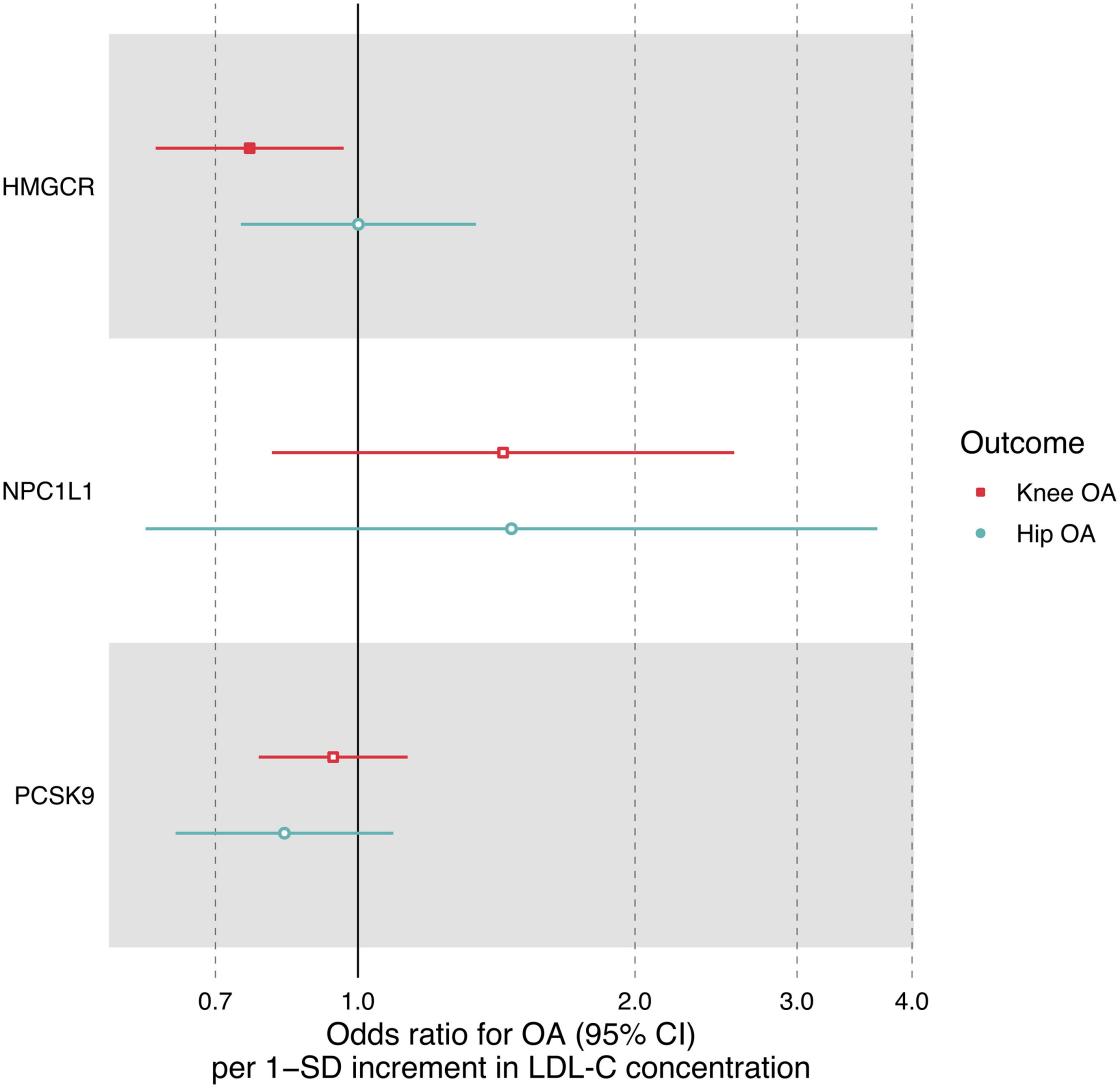
Forest plot comparing causal effect estimates of LDL-C on OA risk restricted to three sets of SNPs within *HMGCR, NPC1L1* and *PCSK9* regions. Odds ratios (ORs) with 95% confidence intervals (CIs) are scaled to 1-SD increment in LDL-C level.

LDL-C increment determined by six SNPs in the *HMGCR* region was significantly associated with a reduced risk of knee OA (OR = 0.76, 95% CI: 0.60–0.96, *P* = 0.024) but not hip OA (OR = 1.00, 95% CI: 0.75–1.34, *P* = 0.994) (Table 3, Figure 3), suggesting that the LDL-C-lowering effect of statins is related to increased risks of knee OA. In contrast, the LDL-C-lowering effect of ezetimibe and evolocumab had no influence on OA risk (Table 3). There was some evidence of heterogeneity across the effects of SNPs in the *NPC1L1* region (*P* = 0.013).

**Table 3:** Estimates of the effect of LDL-C on OA risk using SNPs in specific genes.

**Gene**	**Outcome**	**Number of SNPs**	**OR (95% CI)**	***P*-value**	***P* for heterogeneity**
HMGCR	Knee OA	6	0.76 (0.60–0.96)	0.024	0.610
NPC1L1	Knee OA	5	1.44 (0.81–2.56)	0.219	0.091
PCSK9	Knee OA	7	0.94 (0.78–1.13)	0.512	0.665
HMGCR	Hip OA	6	1.00 (0.75–1.34)	0.994	0.621
NPC1L1	Hip OA	5	1.47 (0.59–3.67)	0.411	0.013
PCSK9	Hip OA	7	0.83 (0.63–1.09)	0.184	0.226

OA, Osteoarthritis; SNP, single nucleotide polymorphisms; HMGCR, 3-hydroxy-3-methylglutaryl-coenzyme A reductase; NPC1L1, Niemann-Pick C1-Like 1; PCSK9, proprotein convertase subtilisin/kexin type 9.

## Discussion

We performed this two-sample MR study to investigate the effects of blood lipids and cholesterol-lowering agents on the risk of knee and hip OA. We found that an increase in LDL-C levels was associated with reduced risks of both knee and hip OA. Multivariate MR analysis proved that this effect was independent of HDL-C level, TG level, and BMI. There was some evidence (from IVW and MR-PRESSO method) that TG increment was associated with reduced risks of hip OA; however, this was not reproduced in the multivariate MR. Another important finding was that the genetically proxied LDL-C-lowering effect of statins was related to increased risks of knee OA but not hip OA. In contrast, the genetically proxied effects of ezetimibe and evolocumab had no influence on OA risk.

In the current study, we performed two-sample MR analyses using different methods under different assumptions, including MR-PRESSO and multivariate MR, which yielded consistent causal effect estimates. Previous studies also investigated the effect of increased LDL-C levels on OA risk using MR analyses (26–28). However, several major differences existed between these existing studies and the current study in terms of analyses and results. Hindy et al. (26) conducted one- and two-sample MR studies based on the Malmö Diet and Cancer Study (MDCS) cohort, which included ~30,000 adults. They found that increased LDL-C levels were associated with reduced overall OA risk (OR = 0.83). Gene-specific subgroup analysis revealed a trend toward reduced OA risk using SNPs within the *HMGCR* gene, but this did not reach statistical significance. However, their sample size was lower than that of the current study. In addition, they did not evaluate site-specific OA risk (26) unlike the present study, wherein we showed the different effects of statin on knee and hip OA. Gill et al. (27) performed a two-sample MR analysis and reported OR estimate for OA risk per 1-SD increment of LDL-C similar to our study (OR = 0.94); however, they did not adjust for other lipids, which could be potential sources of pleiotropy. In addition, they did not investigate site- and gene-specific OA risk. Recently, Meng et al. (28) conducted a two-sample MR analysis and demonstrated that LDL-C increment was associated with reduced risks of both knee OA (OR = 0.899) and hip OA (OR = 0.870). However, they used the same database to estimate SNP-exposure and SNP-outcome association, which could introduce bias in the two-sample MR owing to significant sample overlap (29). Therefore, we believe that our study provides a more robust and specific estimate of the causal effect than previous studies.

Although LDL-C plays a critical role in the pathogenesis of atherosclerosis, its role in OA has received relatively little attention. Since both obesity and hyperlipidemia are manifestations of metabolic syndrome and obesity is a well-recognized risk factor for OA, it is natural to assume that increased LDL-C is also a risk factor for OA (30). Evidence from animal experiments also supports this assumption. In a hyperlipidemic mouse model, Gierman et al. (31) found that a high-cholesterol diet could lead to the development of both OA and atherosclerosis. Interestingly, administration of atorvastatin can suppress the development of both OA and atherosclerosis, whereas ezetimibe only has an effect on atherosclerosis. It was found that lipid deposits in osteoarthritic cartilage and chondrocytes at an early stage of OA, which may trigger the development of OA (32). In addition, oxidized LDL participates in cartilage destruction by activating synovial cells, thereby promoting the release of growth factors and proinflammatory cytokines (30). Nevertheless, our MR results provide an alternative hypothesis that genetically predicted lower LDL-C levels are associated with increased risks of OA. Further research is warranted to explain the discrepancies between animal and human genetic studies.

In line with the effect of LDL-C on OA risk, our MR results suggested that the LDL-C-lowering effect of statins increased the risk of knee OA. Many observational studies have investigated the association between statin use and OA risk; however, conflicting results have been reported (33–40). In a prospective cohort study of 5,674 women, Beattie et al. (33) found that statin use was associated with increased risks of incidental hip OA but not with the progression of hip OA. Eymard et al. (34) performed a *post-hoc* analysis of 336 patients from the SEKOIA trial and found an independent association between statin use and radiological progression of knee OA (OR = 1.49, *P* = 0.010) after adjusting for potential confounding factors. Makris et al. (35) conducted a 1:1 propensity score matching study that included 6,728 statin users and 6,728 non-users. They concluded that statin use led to an increased risk of non-traumatic arthritis (OR = 1.17, 95% CI: 1.09–1.25). In contrast, Clockaerts et al. (36) conducted a prospective cohort study of 2,921 participants and revealed that statin use led to a 50% reduction in overall knee OA progression, as assessed using the Kellgren and Lawrence score. Haj-Mirzaian et al. (37) conducted a retrospective cohort study stratifying participants based on the existence of Heberden nodes (HNs) and found a protective effect of statin use on the progression of radiographic knee OA in HN-positive participants. Other studies have found no effect of statin use on the risk or progression (38–40). A recent meta-analysis of observational studies found high heterogeneity among studies on the effect of statins on the progression of OA (7). Nevertheless, observational studies are inevitably affected by confounding factors, and more importantly, by indication bias (41), which has been well-discussed in studies evaluating statin use and colorectal cancer risk (42). Because of indication bias, observational studies may falsely show a protective effect of statins if hyperlipidemia is related to a lower risk of the disease, which is exactly the current situation since we proved that LDL-C increment was associated with reduced risks of knee and hip OA. According to Mendel's law of inheritance, alleles obtained by individuals in an SNP are random with respect to potential confounding factors. Using SNPs as instrumental variables, MR studies can mimic the effect of randomized controlled trials (43) and can thus provide causal effect estimation closer to the real situation. In addition to statins, ezetimibe and evolocumab are commonly used LDL-C-lowering drugs. However, to the best of our knowledge, no previous study has reported its effects on OA. Further studies need to compare the effects of statins, ezetimibe, and evolocumab on OA, which may overcome the potential indication bias of previous observational studies (44).

The limitations of this study are as follows. First, the MR methodology requires the absence of horizontal pleiotropy, and instrumental variables affect outcomes only through their effect on exposure. In the current study, potential pleiotropy may be due to the effect of instrumental SNPs on other lipids and body weight. Nevertheless, we conducted sensitivity MR analyses using different methods under different assumptions, including MR-Egger regression, which showed no evidence of directional horizontal pleiotropy. We also performed multivariate MR wherein all the analyses yielded similar causal effect estimates. Second, the analyses were conducted based on European ancestry. Therefore, these results may not be applicable to other populations as well. Thirdly, estimates of causal effects were reported as OR per one SD increase in LDL-C, therefore, we could not assess the actual dose-response effect of increasing LDL-C levels and risk of OA. Fourthly, sex may modify the correlation between blood lipid and OA. Future study may stratify male and female individuals to identify this effect. Finally, we used SNPs within *HMGCR, NPC1L1*, and *PCSK9* to mimic the LDL-lowering effect of statins, ezetimibe, and evolocumab. There may be differences between the genetically proxied effect and the real drug effect. In addition, we could not compare the effects of different types of statins.

## Conclusions

In conclusion, our MR study suggests that genetic predisposition to higher blood LDL-C levels may decrease the risk of both knee and hip OA. This effect was independent of HDL-C level, TG level, and BMI. The genetically proxied LDL-C-lowering effects of statins may increase the risk of knee OA but not hip OA. Further studies are needed to reveal the mechanisms underlying the effect of LDL-C and statin on OA and its potential role in treating and preventing OA.

## Data availability statement

Publicly available datasets were analyzed in this study. This data can be found here: https://www.mrbase.org/.

## Ethics statement

Ethical review and approval was not required for the study on human participants in accordance with the Local Legislation and Institutional requirements. The patients/participants provided their written informed consent to participate in this study.

## Author contributions

ZW and YZ designed the study. ZW and ML performed statistical analyses and wrote the manuscript. All authors were involved in results interpreting and were involved in revising the article and approved the final version to be published.

## Conflict of interest

The authors declare that the research was conducted in the absence of any commercial or financial relationships that could be construed as a potential conflict of interest.

## Publisher's note

All claims expressed in this article are solely those of the authors and do not necessarily represent those of their affiliated organizations, or those of the publisher, the editors and the reviewers. Any product that may be evaluated in this article, or claim that may be made by its manufacturer, is not guaranteed or endorsed by the publisher.

## References

[1] March L Cross M Lo C Arden N Gates L Leyland K. Osteoarthritis: a serious disease. OARSI org. (2016) 37:1–3.

[2] Felson DT. Osteoarthritis of the knee. Clinical practice. New Eng J Med. (2006) 354:841–48. 10.1056/NEJMcp051726 16495396

[3] Heidari B Babaei M Yosefghahri B. Prevention of osteoarthritis progression by statins, targeting metabolic and inflammatory aspects: a review. Medit J Rheumatol. (2021) 32:227. 10.31138/mjr.32.3.227 34964026 PMC8693300

[4] Baudart P Louati K Marcelli C Berenbaum F Sellam J. Association between osteoarthritis and dyslipidaemia: a systematic literature review and meta-analysis. RMD open. (2017) 3:e000442. 10.1136/rmdopen-2017-000442 29435358 PMC5706481

[5] Brouwers H von Hegedus J Toes R Kloppenburg M Ioan-Facsinay A. Lipid mediators of inflammation in rheumatoid arthritis and osteoarthritis. Best Pract Res Clin Rheumatol. (2015) 29:741–55. 10.1016/j.berh.2016.02.003 27107510

[6] Fulcher J O'Connell R Voysey M Emberson J Blackwell L Mihaylova B. Efficacy and safety of LDL-lowering therapy among men and women: meta-analysis of individual data from 174,000 participants in 27 randomised trials. Lancet. (2015) 385:1397–405. 10.1016/s0140-6736(14)61368-4 25579834

[7] Wang J Dong J Yang J Wang Y Liu J. Association between statin use and incidence or progression of osteoarthritis: meta-analysis of observational studies. Osteoarth Cartil. (2020) 28:1170–9. 10.1016/j.joca.2020.04.007 32360737

[8] Nurmohamed NS Navar AM Kastelein JJ. New and emerging therapies for reduction of LDL-cholesterol and apolipoprotein B: JACC focus seminar 1/4. J Am Coll Cardiol. (2021) 77:1564–75. 10.1016/j.jacc.2020.11.079 33766264

[9] Skrivankova VW Richmond RC Woolf BAR Davies NM Swanson SA VanderWeele TJ. Strengthening the reporting of observational studies in epidemiology using mendelian randomisation (STROBE-MR): explanation and elaboration. Bmj. (2021) 375:n2233. 10.1136/bmj.n2233 34702754 PMC8546498

[10] Willer CJ Schmidt EM Sengupta S Peloso GM Gustafsson S Kanoni S. Discovery and refinement of loci associated with lipid levels. Nat Genet. (2013) 45:1274. 10.1038/ng.2797 24097068 PMC3838666

[11] Tachmazidou I Hatzikotoulas K Southam L Esparza-Gordillo J Haberland V Zheng J. Identification of new therapeutic targets for osteoarthritis through genome-wide analyses of UK Biobank data. Nat Genet. (2019) 51:230–6. 10.1038/s41588-018-0327-1 30664745 PMC6400267

[12] Sudlow C Gallacher J Allen N Beral V Burton P Danesh J. UK biobank: an open access resource for identifying the causes of a wide range of complex diseases of middle and old age. PLoS Med. (2015) 12:e1001779. 10.1371/journal.pmed.1001779 25826379 PMC4380465

[13] Consortium a Collaborators a. Identification of new susceptibility loci for osteoarthritis (arcOGEN): a genome-wide association study. The Lancet. (2012) 380:815–23. 10.1016/S0140-6736(12)60681-3 22763110 PMC3443899

[14] Bowden J Davey Smith G Haycock PC Burgess S. Consistent estimation in Mendelian randomization with some invalid instruments using a weighted median estimator. Genet Epidemiol. (2016) 40:304–14. 10.1002/gepi.21965 27061298 PMC4849733

[15] Bowden J Davey Smith G Burgess S. Mendelian randomization with invalid instruments: effect estimation and bias detection through Egger regression. Int J Epidemiol. (2015) 44:512–25. 10.1093/ije/dyv080 26050253 PMC4469799

[16] Hemani G Tilling K Davey Smith G. Orienting the causal relationship between imprecisely measured traits using GWAS summary data. PLoS Genet. (2017) 13:e1007081. 10.1371/journal.pgen.1007081 29149188 PMC5711033

[17] Verbanck M Chen CY Neale B Do R. Widespread pleiotropy confounds causal relationships between complex traits and diseases inferred from Mendelian randomization. (2017) 3:7552. 10.1101/157552

[18] Yengo L Sidorenko J Kemper KE Zheng Z Wood AR Weedon M. Meta-analysis of genome-wide association studies for height and body mass index in ~7,00,000 individuals of European ancestry. Hum Mol Genet. (2018) 27:3641–9. 10.1093/hmg/ddy271 30124842 PMC6488973

[19] Ference BA Majeed F Penumetcha R Flack JM Brook RD. Effect of naturally random allocation to lower low-density lipoprotein cholesterol on the risk of coronary heart disease mediated by polymorphisms in NPC1L1, HMGCR, or both: a 2 × 2 factorial Mendelian randomization study. J Am Coll Cardiol. (2015) 65:1552–61. 10.1016/j.jacc.2015.02.020 25770315 PMC6101243

[20] Ference BA Robinson JG Brook RD Catapano AL Chapman MJ Neff DR. Variation in PCSK9 and HMGCR and risk of cardiovascular disease and diabetes. N Engl J Med. (2016) 375:2144–53. 10.1056/NEJMoa1604304 27959767

[21] Zheng J Brion MJ Kemp JP Warrington NM Borges MC Hemani G. The effect of plasma lipids and lipid-lowering interventions on bone mineral density: a mendelian randomization study. J Bone Miner Res. (2020 J) 35:1224–35. 10.1002/jbmr.3989 32163637

[22] Liu G Shi M Mosley JD Weng C Zhang Y Lee MTM. A Mendelian Randomization Approach Using 3-HMG-Coenzyme-A reductase gene variation to evaluate the association of statin-induced low-density lipoprotein cholesterol lowering with noncardiovascular disease phenotypes. JAMA Netw Open. (2021) 4:e2112820. 10.1001/jamanetworkopen.2021.12820 34097045 PMC8185593

[23] Gormley M Yarmolinsky J Dudding T Burrows K Martin RM Thomas S. Using genetic variants to evaluate the causal effect of cholesterol lowering on head and neck cancer risk: a Mendelian randomization study. PLoS Genet. (2021 A) 17:e1009525. 10.1371/journal.pgen.1009525 33886544 PMC8096036

[24] Yavorska OO Burgess S. MendelianRandomization: an R package for performing Mendelian randomization analyses using summarized data. Int J Epidemiol. (2017) 46:1734–9. 10.1093/ije/dyx034 28398548 PMC5510723

[25] Hemani G Zheng J Elsworth B Wade KH Haberland V Baird D. The MR-Base platform supports systematic causal inference across the human phenome. Elife. (2018) 7:34408. 10.7554/eLife.34408 29846171 PMC5976434

[26] Hindy G Åkesson KE Melander O Aragam KG Haas ME Nilsson PM. Cardiometabolic polygenic risk scores and osteoarthritis outcomes: a Mendelian randomization study using data from the Malmö diet and cancer study and the UK biobank. Arthritis Rheumatol. (2019 J) 71:925–34. 10.1002/art.40812 30615301 PMC6563114

[27] Gill D Karhunen V Malik R Dichgans M Sofat N. Cardiometabolic traits mediating the effect of education on osteoarthritis risk: a Mendelian randomization study. Osteoarthritis and cartilage. (2021) 29:365–71. 10.1016/j.joca.2020.12.015 33422704 PMC7955282

[28] Meng H Jiang L Song Z Wang F. Causal associations of circulating lipids with osteoarthritis: a bidirectional mendelian randomization study. Nutrients. (2022) 14:1327. 10.3390/nu14071327 35405941 PMC9000847

[29] Pierce BL Burgess S. Efficient design for Mendelian randomization studies: subsample and 2-sample instrumental variable estimators. Am J Epidemiol. (2013) 178:1177–84. 10.1093/aje/kwt084 23863760 PMC3783091

[30] de Munter W van der Kraan PM van den Berg WB van Lent PL. High systemic levels of low-density lipoprotein cholesterol: fuel to the flames in inflammatory osteoarthritis? Rheumatology. (2016 J) 55:16–24. 10.1093/rheumatology/kev270 26231344

[31] Gierman LM Kühnast S Koudijs A Pieterman EJ Kloppenburg M van Osch GJ. Osteoarthritis development is induced by increased dietary cholesterol and can be inhibited by atorvastatin in APOE^*^3LeidenCETP mice–a translational model for atherosclerosis. Ann Rheum Dis. (2014 M) 73:921–7. 10.1136/annrheumdis-2013-203248 23625977

[32] Zhang K Ji Y Dai H Khan AA Zhou Y Chen R Jiang Y Gui J. High-density lipoprotein cholesterol and apolipoprotein A1 in synovial fluid: potential predictors of disease severity of primary knee osteoarthritis. Cartilage. (2021) 13(1_suppl):1465s−73s. 10.1177/19476035211007919 33870758 PMC8808802

[33] Beattie MS Lane NE Hung YY Nevitt MC. Association of statin use and development and progression of hip osteoarthritis in elderly women. J Rheumatol. (2005 J) 32:106–10. 15630734

[34] Eymard F Parsons C Edwards MH Petit-Dop F Reginster JY Bruyère O. Statin use and knee osteoarthritis progression: Results from a post-hoc analysis of the SEKOIA trial. Joint Bone Spine. (2018 O) 85:609–14. 10.1016/j.jbspin.2017.09.014 29037516 PMC5858762

[35] Makris UE Alvarez CA Mortensen EM Mansi IA. Association of Statin use with increased risk of musculoskeletal conditions: a retrospective cohort study. Drug Saf. (2018 O) 41:939–50. 10.1007/s40264-018-0682-y 29797239 PMC6143406

[36] Clockaerts S Van Osch GJ Bastiaansen-Jenniskens YM Verhaar JA Van Glabbeek F Van Meurs JB. Statin use is associated with reduced incidence and progression of knee osteoarthritis in the Rotterdam study. Ann Rheum Dis. (2012 M) 71:642–7. 10.1136/annrheumdis-2011-200092 21989540

[37] Haj-Mirzaian A Mohajer B Guermazi A Conaghan PG Lima JAC Blaha MJ. Statin use and knee osteoarthritis outcome measures according to the presence of heberden nodes: results from the osteoarthritis initiative. Radiology. (2019 N) 293:396–404. 10.1148/radiol.2019190557 31502936

[38] Michaëlsson K Lohmander LS Turkiewicz A Wolk A Nilsson P Englund M. Association between statin use and consultation or surgery for osteoarthritis of the hip or knee: a pooled analysis of four cohort studies. Osteoarthritis Cartilage. (2017 N) 25:1804–13. 10.1016/j.joca.2017.07.013 28756279

[39] Peeters G Tett SE Conaghan PG Mishra GD Dobson AJ. Is statin use associated with new joint-related symptoms, physical function, and quality of life? Results from two population-based cohorts of women. Arthritis Care Res. (2015 J) 67:13–20. 10.1002/acr.22389 24964875

[40] Riddle DL Moxley G Dumenci L. Associations between statin use and changes in pain, function and structural progression: a longitudinal study of persons with knee osteoarthritis. Ann Rheum Dis. (2013 F) 72:196–203. 10.1136/annrheumdis-2012-202159 23172752

[41] Bosco JL Silliman RA Thwin SS Geiger AM Buist DS Prout MN. A most stubborn bias: no adjustment method fully resolves confounding by indication in observational studies. J Clin Epidemiol. (2010 J) 63:64–74. 10.1016/j.jclinepi.2009.03.001 19457638 PMC2789188

[42] Mamtani R Lewis JD Scott FI Ahmad T Goldberg DS Datta J. Disentangling the association between statins, cholesterol, and colorectal cancer: a nested case-control study. PLoS Med. (2016 A) 13:e1002007. 10.1371/journal.pmed.1002007 27116322 PMC4846028

[43] Holmes MV Ala-Korpela M Smith GD. Mendelian randomization in cardiometabolic disease: challenges in evaluating causality. Nat Rev Cardiol. (2017 O) 14:577–90. 10.1038/nrcardio.2017.78 28569269 PMC5600813

[44] Yoshida K Solomon DH Kim SC. Active-comparator design and new-user design in observational studies. Nat Rev Rheumatol. (2015 J) 11:437–41. 10.1038/nrrheum.2015.30 25800216 PMC4486631

