# Supplementary material for: Effect of blood lipids and lipid-lowering therapies on osteoarthritis risk: A Mendelian randomization study
Source: Front Med (Lausanne). 2022 Nov 11;9:990569. doi: 10.3389/fmed.2022.990569 (PMC9691771; doi:10.3389/fmed.2022.990569)
Supplement: Supplementary file 1 [file Data_Sheet_1.DOCX]

**Fig. S1** Funnel plots of MR analyses for the causal effect of LDL-C on knee OA risk.

**Fig. S2** Funnel plots of MR analyses for the causal effect of LDL-C on hip OA risk.

**Fig. S3** Funnel plots of MR analyses for the causal effect of HDL-C on knee OA risk.

**Fig. S4** Funnel plots of MR analyses for the causal effect of HDL-C on hip OA risk.

**Fig. S5** Funnel plots of MR analyses for the causal effect of TG on knee OA risk.

**Fig. S6** Funnel plots of MR analyses for the causal effect of TG on hip OA risk.
